# Monoclonal Antibodies Generated against Glycoconjugates Recognize Chemical Linkers

**DOI:** 10.3390/antib9030048

**Published:** 2020-09-15

**Authors:** Jessica Ramadhin, Vanessa Silva-Moraes, Thomas Norberg, Donald Harn

**Affiliations:** 1 Department of Infectious Diseases, College of Veterinary Medicine, University of Georgia, Athens, GA 30602, USA; jramadhin@uga.edu (J.R.); vmoraes@uga.edu (V.S.-M.); 2 Department of Biochemistry and Organic Chemistry, Uppsala University, 752 36 Uppsala, Sweden; thomas.norberg@kemi.uu.se

**Keywords:** glycans, glycoconjugates, monoclonal antibodies, human milk oligosaccharides, lacto-N-fucopentaose III, lacto-N-neotetraose, Lewis^x^ antigen, acetylphenylenediamine

## Abstract

Monoclonal antibodies (mAbs) that recognize glycans are useful tools to assess carbohydrates’ structure and function. We sought to produce IgG mAbs to the human milk oligosaccharide (HMO), lacto-N-fucopentaose III (LNFPIII). LNFPIII contains the Lewis^x^ antigen, which is found on the surface of schistosome parasites. mAbs binding the Lewis^x^ antigen are well-reported in the literature, but mAbs recognizing HMO structures are rare. To generate mAbs, mice were immunized with LNFPIII-DEX (P3DEX) plus CpGs in VacSIM^®^, a novel vaccine/drug delivery platform. Mice were boosted with LNFPIII-HSA (P3HSA) plus CpGs in Incomplete Freund’s Adjuvant (IFA). Splenocytes from immunized mice were used to generate hybridomas and were screened against LNFPIII conjugates via enzyme-linked immunosorbent assay (ELISA). Three positive hybridomas were expanded, and one hybridoma, producing IgG and IgM antibodies, was cloned via flow cytometry. Clone F1P2H4D8D5 was selected because it produced IgG1 mAbs, but rescreening unexpectedly showed binding to both LNFPIII and lacto-N-neotetraose (LNnT) conjugates. To further assess the specificity of the mAb, we screened it on two glycan microarrays and found no significant binding. This finding suggests that the mAb binds to the acetylphenylenediamine (APD) linker-spacer structure of the conjugate. We present the results herein, suggesting that our new mAb could be a useful probe for conjugates using similar linker spacer structures.

## 1. Introduction

Glycans are considered core biological building blocks. Glycans are ubiquitous in nature and exert their effects via their own properties or via the modification of proteins and lipids. Their presence on cell surfaces provide strength and protection, and they also serve as ligands for receptors (i.e., selectins, galectins, C-type lectins, Siglecs, etc.) to modulate signaling [1]. Glycans are crucial for communication between microbial and more complex species (i.e., plants, animals, humans) and are heavily involved in the innate and adaptive immune responses. The biological roles of glycans have been extensively reviewed in Varki (2016) [2].

The field of glycobiology lacks well-developed tools that facilitate a functional analysis. In terms of structure, glycans are more complex than nucleic acids and proteins because of the assortment of known monosaccharides and their ability to be linked in various numbers and fashions (i.e., branched, anomeric) [3]. In this regard, glycan-binding proteins (GBPs) have become fundamental for assessing carbohydrates’ structure and function. Currently, the two most widely used tools for the quantification and/or localization of specific glycans include lectins and glycan-binding antibodies [4]. Various plant and animal lectins have been well-characterized in terms of sequences and binding specificities and are typically available at a low cost [5,6]. Microarrays containing lectins have been developed, being simpler and more sensitive than traditional mass spectrometry (MS) methods [7,8,9,10,11]. The drawback is that lectins bind their determinants with differing affinities that depend on the glycan in question. For example, concanvalin A (ConA) recognizes oligomannose-type-N-glycans with a much higher affinity than more complex biantennary N-glycans [12].

In contrast to lectins, GBPs bind to specific determinants and do not discriminate between O-glycans, N-glycans, or glycolipids [12]. Several approaches have been utilized to generate glycan-binding antibodies and include generating hybridomas using the splenocytes of mice immunized with whole cells or glycan-protein conjugates or mice that have been infected with pathogens [13]. A specific example of this is the generation of the anti-glycan mAb (E.5) from mice immunized with living schistosome eggs or soluble egg antigens (SEA). The E.5 mAb binds the Lewis^x^ trisaccharide, α-L-Fuc-(1→3)-[β-D-Gal-(1→4)]-D-GlcNAc, present on the surface of schistosome eggs, adult tissues, and cancerous tumors [14,15,16,17]. E.5 also binds to the human milk oligosaccharide (HMO) and pre-implantation antigen, lacto-N-fucopentaose III (LNFPIII; β-D-Gal-(1→4)-[α-L-Fuc-(1→3)]-β-D-GlcNAc-(1→3)-β-D-Gal-(1→4)-D-Glc) [17]. A drawback of the E.5. mAb is that it is IgM, which makes it difficult to purify [18]. Herein, we sought to produce IgG mAbs against LNFPIII, which contains the Lewis^x^ antigen. mAbs against the Lewis^x^ antigen are well-reported in the literature, but mAbs recognizing HMO structures are rare [13,17,19]. Previous studies suggest that Lewis^x^ must be presented on adjacent molecules or in a multimeric form to bind to or activate cells [20]. HMOs, such as LNFPIII and Lacto-N-neotetraose (LNnT; β-D-Gal-(1→4)-β-D-GlcNAc-(1→3)-β-D-Gal-(1→4)-D-Glc), are detected in human breastmilk in their free form or are attached to proteins and lipids, and do not induce a mAb response to our knowledge [21].

Here, we report the development and characterization of a novel IgG mAb (F1P2H4D8D5) generated from the splenocytes of mice immunized with LNFPIII glycan conjugates. LNFPIII conjugates are composed of 10–12 molecules of LNFPIII conjugated to a 40 kDa dextran (P3DEX) or to human serum albumin (P3HSA) via an acetylphenylenediamine (APD) linker. The combined use of a carrier (DEX or HSA) and this linker method increases the concentration of LNFPIII in the conjugates and allows each molecule to rotate in space to bind to cellular receptors. Unlike free LNFPIII, these LNFPIII conjugates have been shown to act on B cells, macrophages, dendritic cells, hepatocytes, and adipocytes in vivo [22,23,24,25,26].

The initial screening of mAb F1P2H4D8D5 showed binding to LNFPIII conjugates and to conjugates containing the structurally similar tetrasaccharide, LNnT. LNnT conjugates are also conjugated to a 40 kDa dextran (NTDEX) or to human serum albumin (NTHSA). The results of additional screens suggested that the mAb might be binding to the linker spacer of the conjugate and this possibility was partially confirmed when the mAb failed to bind to any glycan structures on both human milk and general glycan structure microarrays. Since APD linkers are used to produce a wide array of conjugates, an anti-APD mAb is valuable to the research community as a probe for any structures produced using the same linker [27,28]. The advantage of this IgG mAb is that it could be used in laboratory settings as a functional probe for chemically synthesized glycoconjugates.

## 2. Materials and Methods

### 2.1. Chemicals and Parasite Extracts

LNFPIII was synthesized by Dr. George Wang (Georgia State University, Atlanta, GA, USA). LNnT was synthesized by Neose Technologies, Inc. LNFPIII and LNnT were sent to Dr. Thomas Norberg (Uppsala University, Suppsala, Sweden) for conjugation to carriers, dextran from Leuconostoc mesenteroides (Sigma Aldrich, St. Louis, MO, USA, Cat. No. D1662), or human serum albumin (Millipore Sigma, St. Louis, MO, USA, Cat. No. A3782) using proprietary APD linker-spacers. On average, each conjugate had 10–12 HMO monomers per 40 kDa dextran or HSA.

Soluble schistosome egg antigen (SEA) was prepared as described previously [14]. Briefly, Swiss Webster mice were infected with Schistosoma mansoni (PR strain) cercariae obtained from infected snails provided by BEI Resources, the NIAID Schistosomiasis Resource Center. Mice were infected with 100–150 infectious cercariae of S. mansoni via intraperitoneal injection. 7–8 weeks post-infection, parasite eggs were harvested from livers. SEA was prepared by homogenizing purified eggs in phosphate-buffered saline (PBS), pH 7.4, for 1 h at 4 °C. The egg homogenate was then centrifuged at 15,000× *g* for 1 h at 4 °C, and the supernatant was collected as SEA. Protein was quantified via the Pierce™ BCA Protein Assay Kit (Thermofisher Scientific, Waltham, MA, USA, Cat. No. 23227) and stored at −80 °C until use.

Schistosomula membrane protein (SMP) was prepared as described previously [14]. In brief, S. mansoni cercariae were mechanically transformed into schistosomula using a Vortex mixer. Parasite bodies were then separated from tails via centrifugation on a Percoll gradient and incubated for 24 h in Corning™ Cellgro™ RPMI 1640 (Corning™, Cat. No. 10-040-CV) enriched with 5% Corning™ Regular Fetal Bovine Serum (FBS) (Corning™, Cat. No. 35010CV). Surface extracts of schistosomula were prepared by incubating mechanical schistomula in 50 mM phosphate buffer, pH 8.0, containing 4 mM deoxycholate in detergent solution on ice for 30 min (100 parasites/µL). Soluble SMP was collected via centrifugation of the detergent extract at 15,000× *g* for 1 h at 4 °C. Deoxycholate was removed from SMP by desalting on Pierce™ Polyacrylamide Spin Desalting Columns, 7K MWCO, 0.7 mL (ThermoFisher Scientific, Waltham, MA, USA, Cat. No. 89849). Protein was quantified via the Pierce™ BCA Protein Assay Kit (Thermofisher Scientific, Waltham, MA, USA, Cat. No. 23227) and stored at −80 °C until use.

### 2.2. Mouse Immunizations

Glycoconjugates were dissolved in 0.9% sterile saline and stored at −80 °C until use. 200 µg of P3DEX plus 50 µg of CpG ODN 1826 (InvivoGen, San Diego, CA, USA, Cat. No. tlrl-1826) were added to 1% *w*/*v* VacSIM™ (Vaccine Self-Assembling Immune Matrix) [29], then injected subcutaneously into 4 BALB/c mice at D1. At D21, mice received a second dose containing 200 µg of P3HSA plus 50 µg of CpG ODN 1826 and an equivalent volume of incomplete Freund’s adjuvant. A third dose of P3HSA plus 50 µg of CpG ODN 1826 and an equivalent volume of incomplete Freund’s adjuvant was injected intraperitoneally three days prior to the removal of spleens (D42). Mice were sacrificed on D45, and the spleens were collected for cell fusion and the production of hybridomas, as described in Section 2.3. Serum was collected at D0, D21, and D35 for screening against LNFPIII conjugates.

### 2.3. Cell Fusion, Hybridoma Selection, & Screening

Mice were sacrificed on D45 for spleen removal and the preparation of splenocytes. Red blood cells (RBCs) were lysed by adding 2 mL of RBC Lysis Buffer (Sigma, St. Louis, MO, USA, Cat. No. R7757) and incubating for 3 min, followed by two washes in HyClone™ Dulbecco’s Modified Eagles Medium (DMEM) (HyClone™, Cat. No. SH30081.01). Sp2/0-Ag14 cells (ATCC^®^ CRL-1581™) were grown to 100% confluence in DMEM containing 20% FBS + 8 mM L-glutamine + 100 µg/mL penn/strep at 37 °C in 5% CO_2_. Splenocytes were mixed with Sp2/0-Ag14 cells at a ratio of 8:1 splenocytes per Sp2/0-Ag14 cell and then pelleted by centrifugation. The supernatant was removed, and cells were dislodged by thwacking the tube. Cell fusion was induced by adding 0.7 mL of 50% polyethylene glycol (PEG, MW 1500; Sigma, St. Louis, MO, USA, Cat. No.) for 1 min at 37 °C. Cells were pelleted, the supernatant was removed, and cells were gently resuspended by slow addition of 7 mL of warm (37 °C) DMEM. Then, cells were diluted with an additional 43 mL of hybridoma HAT media (DMEM supplemented with Gibco™ HAT Supplement (50X) (Gibco™, Cat. No. LS21060017)) + 15% FBS + 1% oxaloacetate-pyruvate-insulin (OPI) (Millipore Sigma, St. Louis, MO, USA, Cat. No. O5003-1VL) + 100 µg/mL penn/strep and plated into 96-well plates at 100 µL/well. 7D post-fusion, 100 µL/well of hybridoma HT media (DMEM) supplemented with hypoxanthine-thymidine (HT) + 15% FBS + 1% OPI + 100 µg/mL penn/strep was added. Plates were screened for hybridoma growth and the cell supernatants were tested by ELISA against P3DEX. Hybridomas positive for P3DEX and IgG, but not HSA and DEX, were cloned via flow sorting on a MoFlo Astrios Cell Sorter. In brief, cells were stained using a LIVE/DEAD™ Fixable Near-IR Dead Cell Stain Kit (Invitrogen, Waltham, MA, USA, Cat. No. L10119), resuspended in hybridoma HT media, and sorted into a single cell per well. The determination of Ig subtypes was performed with the SBA Clonotyping System-HRP (Southern Biotech, Birmingham, AL, USA, Cat. No. 5300-05) via indirect ELISA, in order to determine the specific immunoglobulin subclass. Positive hybridomas were propagated and stored in liquid nitrogen.

### 2.4. Enzyme-Linked Immunosorbent Assay (ELISA)

Nunc MaxiSorp™ flat-bottom plates (Invitrogen™, Cat. No. 44-2402-21) were coated at 100 µL/well with one of the following: (1) 5 μg/mL DEX, (2) 5 μg/mL HSA, (3) 50 μg/mL P3DEX, (4) 100 μg/mL P3HSA, (5) 50 μg/mL NTDEX, (6) 100 μg/mL NTHSA, (7) 5 μg/mL SEA, or (8) 5 μg/mL SMP in 0.05 M carbonate bicarbonate buffer, pH 9.6, and incubated for 16 h at 4 °C. Plates were then washed 6× in PBS with 0.05% Tween-20 (PBST) and blocked via the addition of 300 µL/well of 2.5% milk in PBST at room temperature (RT) for 2 h. After additional washing, 100 µL/well of mice serum (1:100 in PBS) or hybridoma supernatant was added to the plate and incubated at RT for 2 h. Plates were washed and horseradish peroxidase-conjugated anti-mouse IgG (H+L) and IgM (Roche, Cat. No. 0311693001) or a panel of isotyping antibodies (IgG1, IgG2a, IgG2b, IgG3, and IgA; Southern Biotech, Birmingham, AL, USA, Cat. No. 5300-05) were added as required to the wells at a dilution of 1:4000 in PBST at RT for 1 h. Plates were washed four times, and color was developed using 3,3′,5,5′-tetramethylbenzidine (TMB; Sigma, St. Louis, MO, USA, Cat. No. T0440). ELISA reactions were stopped after 10 min of incubation in the dark at RT with 50 µL of sulfuric acid. The optical density (OD) was determined using the SPECTROstar Nano Microplate Reader at 450/570 nm.

### 2.5. mAb Purification

We selected a hybridoma clone secreting IgG1: mAb F1P2H4D8D5 for further analysis. Hybridomas were grown to 80% confluency in DMEM containing 20% FBS + 8 mM L-glutamine + 100 µg/mL penn/strep at 37 °C in 5% CO_2_. Supernatants were recovered at 100% confluence and stored at 4 °C until purification. In order to isolate IgG antibodies, hybridoma culture supernatants were purified via ammonium sulfate precipitation according to Fishman and Berg (2018), with some modifications [30]. In brief, hybridoma cell culture supernatants were centrifuged at 3000× *g* for 30 min at 4 °C. Supernatants were then slowly mixed with saturated ammonium sulfate for a final *w*/*v* concentration of 25% and incubated at 4 °C overnight. The solution was then centrifuged again at 3000× *g* for 30 min at 4 °C. Saturated ammonium sulfate was then added to reach a final *w*/*v* concentration of 50% and was incubated at 4 °C overnight. The solution then underwent a third round of centrifugation at 3000× *g* for 30 min at 4 °C. The supernatant was discarded, and the remaining pellet was resuspended in 30% of the original volume of 1× PBS. The sample was dialyzed against three changes of 1× PBS using 10 K Slide-A-Lyzer™ cassettes (ThermoFisher Scientific, Waltham, MA, USA, Cat. No. 66456). The protein concentration was determined using a Nanodrop 2000 spectrophotometer. Following ammonium sulfate precipitation, eluted proteins were further purified using HiTrap protein G columns (GE Healthcare Life Sciences, Chicago, IL, USA, Cat. No. 29-0485-81) according to the manufacturer’s protocol. After protein quantification, 2.5 or 5 µg of purified antibody was loaded into two identical 4–20% SDS-PAGE gels. Gels were run for 2 h at 50 V, 3.00 A, 300 W using the PowerPac™ HC High-Current Power Supply (BioRad, Hercules, CA, USA, Cat. No. 1645052). One gel was stained with Coomassie Brilliant Blue R-250 (Bio-Rad, Hercules, CA, USA, Cat. No. 1610436), and the other was transferred to a PVDF membrane after electrophoresis using the Trans-Blot Turbo RTA Mini 0.45 µm LF PVDF Transfer Kit (BioRad, Hercules, CA, USA, Cat. No. 1704274). Gels were transferred using the Trans-Blot Turbo Transfer System (BioRad, Hercules, CA, USA, Cat. No. 1704150) programmed for mixed molecular weight proteins at 2.5 A and 25 V for 7 min. The membrane was blocked at 4 °C for 16 h in 5% milk in tris-buffered saline plus 0.05% Tween (TBST). Subsequently, the membrane was incubated with peroxidase-conjugated anti-mouse IgG and IgM diluted in 3% milk in TBST at RT for 1 h. The membrane was washed 5× and revealed using the ECL Plus Western Blotting Detection System (GE Healthcare, Chicago, IL, USA, Cat. No.), and images were captured using chemiluminscence detection on a Bio-Rad imager.

### 2.6. RAW 264.7 Cell Culture

RAW 264.7 (ATCC^®^ TIB-71™) cells were grown in DMEM plus 10% FBS, 4 mM L-glutamine, and 100 U/mL + 100 µg/mL penn/strep. For the experiments, 3 × 10^5^ cells/well were plated in a 6-well plate. Once 70–80% confluent, cells were stimulated with (1) sterile saline, (2) 50 µg/mL DEX, (3) 50 µg/mL HSA, (4) 50 µg/mL P3DEX, (5) 50 µg/mL NTDEX, (6) 50 µg/mL P3HSA, or (7) 50 µg/mL NTHSA. Cells were stimulated for 60 s, washed, and lysed using NP-40 buffer (ThermoFisher Scientific, Waltham, MA, USA, Cat. No. FNN0021). Protein was quantified using a Nanodrop, and lysates were stored at −80 °C until use.

### 2.7. Characterization of Monoclonal Antibody

mAbs were characterized in terms of: (1) specificity to different antigen targets in ELISAs and Western blots, and (2) a microarray analysis conducted at Harvard’s Center for Functional Glycomics (NCFG). ELISAs were performed according to Section 2.4 with the following modifications. For the characterization of mAb, 100 µL of hybridoma supernatant from clone F1P2H4D8D5 was added to the plate and incubated at RT for 2 h. Plates were washed and Goat Anti-Mouse Ig, Human ads-HRP (Southern Biotech, Birmingham, AL, USA, Cat. No. 1010-05) was added to the wells at a dilution of 1:4000 in PBST at RT for 1 h. For Western blots, 5 or 10 µg of treated RAW 264.7 cell lysates were run on a 4–20% Mini-PROTEAN^®^ TGX™ Precast Protein Gel (BioRad, Hercules, CA, USA, Cat. No. 4561096) and transferred to a PVDF membrane, as described in Section 2.5, with the following modifications. After the protein transfer, the membrane was incubated with peroxidase-conjugated anti-mouse IgG and IgM diluted in 3% milk in TBST at RT for 1 h. For the microarray analysis, purified mAb F1P2H4D8D5 was sent to Harvard’s NCFG, and 200 µg was run on both the Human Milk Glycan and NCFG arrays. The methods and structures present on these arrays are found at https://ncfg.hms.harvard.edu/microarrays and are described in [31].

## 3. Results

**Figure 1:**
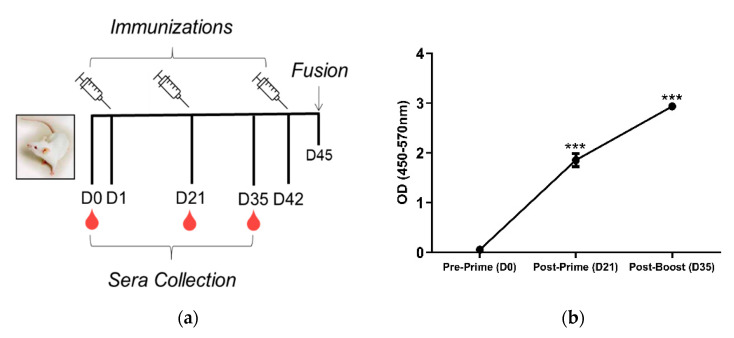
Experimental timeline and anti-P3 conjugate response following immunization. (**a**) BALB/c mice were primed with 200 µg P3DEX plus 50 µg of CpG ODN 1826 delivered in VacSIM™ (Vaccine Self-Assembling Immune Matrix) *w*/*v* on D1 and boosted with 200 µg P3HSA plus 50 µg of CpG ODN 1826 and an equivalent volume of incomplete Freund’s adjuvant on D21 and D42. Blood was collected pre-prime (D0), post-prime (D21), and post-boost (D35) (**b**) Sera collected on D0, D21, and D35 were evaluated via ELISA to evaluate specific antibody responses against LNFPIII conjugates. *** *p* < 0.001 (*t*-test, CI: 95%).

4 BALB/c mice were immunized using the prime-boost regimen depicted in Figure 1a. Mice were primed via the subcutaneous route with 200 µg P3DEX plus 50 µg of CpG ODN 1826 delivered in VacSIM™ (Vaccine Self-Assembling Immune Matrix) *w*/*v* [27]. At D21, mice were boosted with 200 µg of P3HSA plus 50 µg of CpG ODN 1826 and an equivalent volume of incomplete Freund’s adjuvant to enhance the mAb response. Mice were boosted again on D42 using the same formula. Serum was collected on D0, D21, and D35, and screened via ELISA. Anti-P3 conjugate responses were detected in sera post-prime and post-boost. The anti-P3 conjugate immunoglobulin (Ig) levels were significantly higher post-prime and post-boost (*p* < 0.001) compared to the pre-prime levels (Figure 1b).

**Figure 2:**
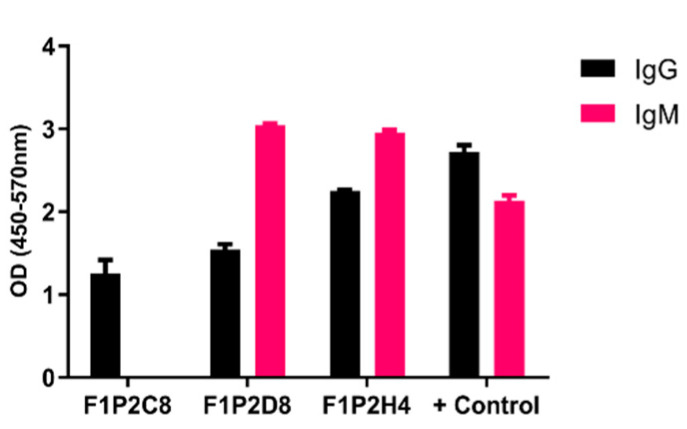
IgG and IgM antibodies produced by hybridomas. Hybridomas were screened 15−30 D post-fusion. Supernatants were screened by ELISA against P3DEX to assess the specific IgG and IgM levels. Hybridomas were positive for P3DEX, but not for the carriers DEX and HSA. Sera from immunized mice were used as a positive control.

**Figure 3:**
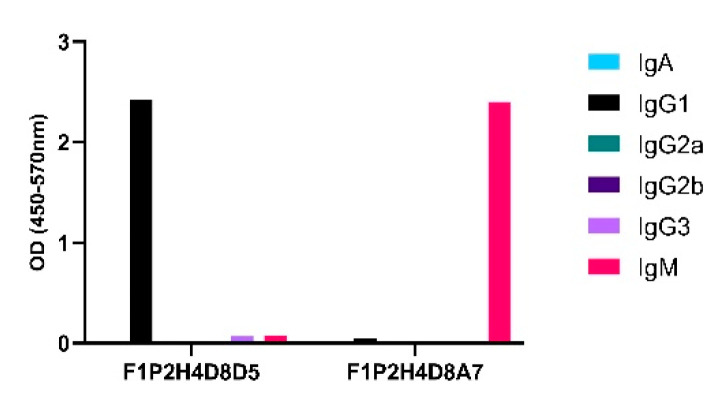
Specific class and subclass of selected clones secreting anti-P3 antibodies. Supernatants from clones were screened via ELISA using P3DEX and the carriers DEX and HSA as the antigenic targets.

Upon sacrifice and 30D post-cellular fusion, 75/384 wells (19.5%) contained hybridomas. The screening of hybridomas generated from one mouse yielded three hybridomas (3/75) producing antibodies to LNFPIII conjugates. We isotyped these hybridomas and determined that F1P2C8 was IgG subclass, while F1P2D8 and F1P2H4 were both IgG and IgM (Figure 2). F1P2H4 was cloned via flow cytometry and screened again, yielding mAbs F1P2H4D8A7 (IgM) and F1P2H4D8D5 (IgG1) (Figure 3). Supernatants from clone F1P2H4D8D5 were picked for purification and subsequent experiments.

**Figure 4:**
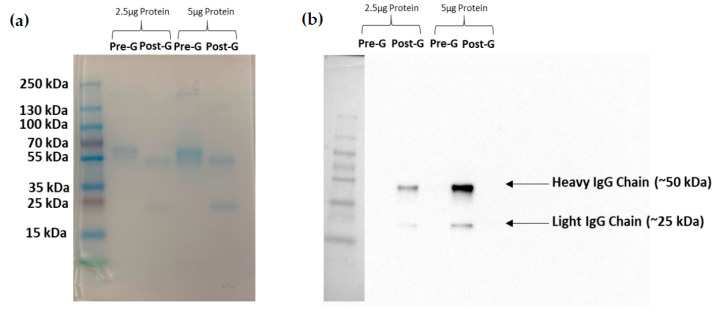
Purification and enrichment of mAb F1P2H4D8D5. mAb was run on two SDS-PAGE gels and was (**a**) stained with Coomassie Blue or (**b**) transferred to a PVDF membrane and probed with horseradish peroxidase-conjugated anti-mouse IgG. The SDS-PAGE stained with Coomassie showed the elimination of contaminating proteins post-G column purification. The membrane showed the enrichment of IgG post-G column purification.

Following ammonium sulfate precipitation and protein G column purification, supernatants from clone F1P2H4D8D5 were run via SDS-PAGE electrophoresis. Table 1 lists the protein concentrations and 260/280 ratios following each purification step. Figure 4a presents an SDS-PAGE gel stained with Coomassie Blue, demonstrating that we were able to eliminate various protein contaminants using protein G column purification. Figure 4b depicts a Western blot of purified F1P2H4D8D5 probed with anti-mouse IgG (H+L). This particular Western blot shows the heavy (~50 kDa) and light (~25 kDa) IgG chains of the purified antibody detected post-protein G column purification. Overall, this demonstrates that we were able to successfully purify and concentrate mAb F1P2H4D8D5.

**Figure 5:**
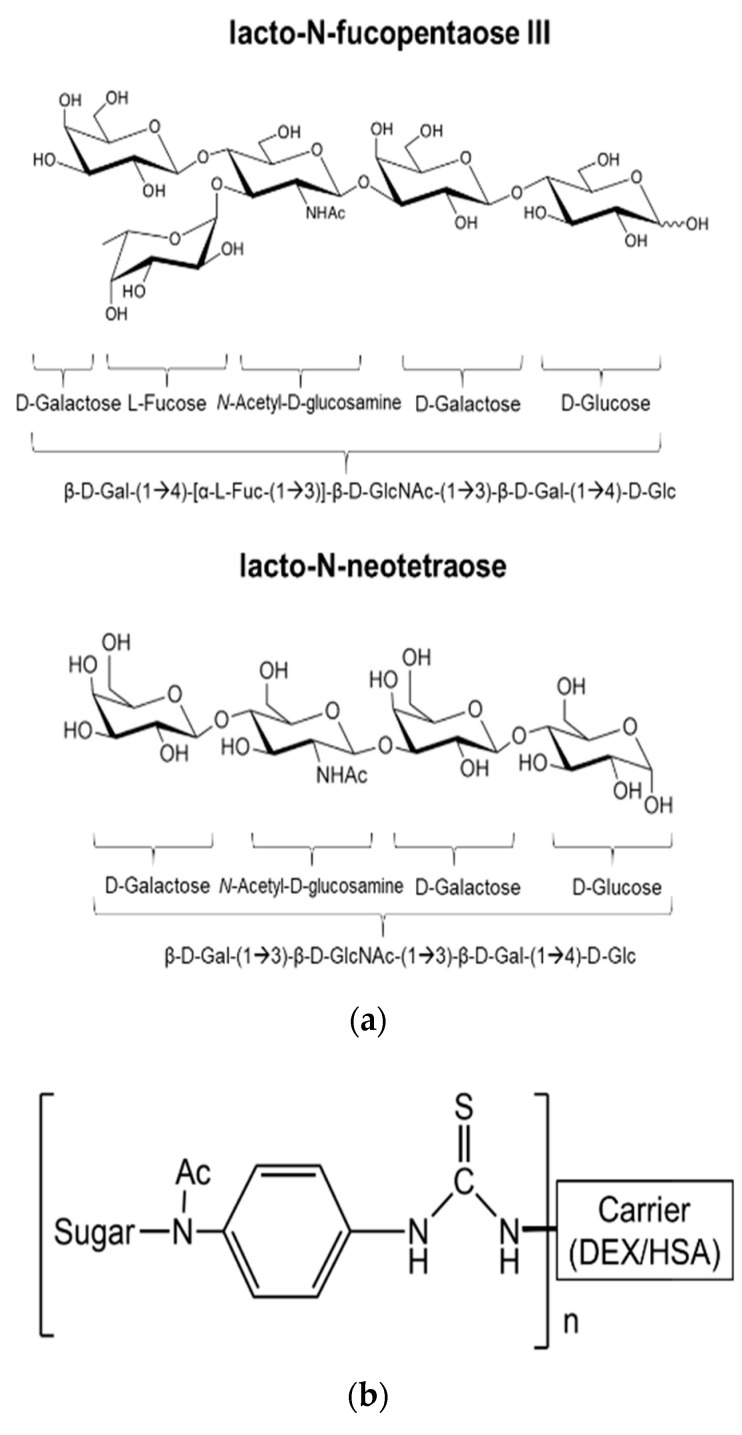
Structure of LNFPIII, LNnT, and conjugates. (**a**) Chemical structure of LNFPIII and LNnT; (**b**) Chemical structure of LNFPIII and LNnT conjugates. 10–12 molecules of LNFPIII or LNnT were conjugated to DEX or HSA via an APD linker.

**Figure 6:**
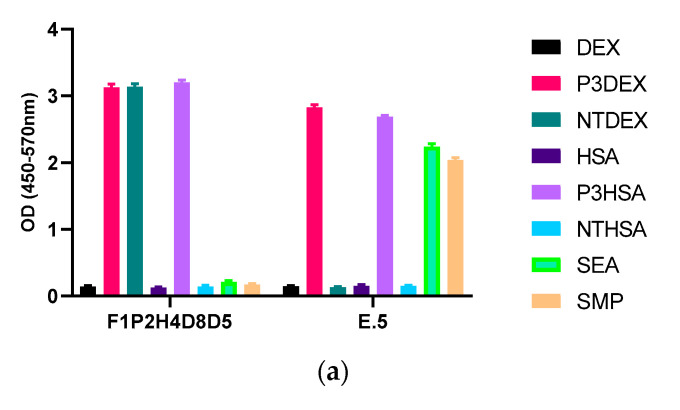

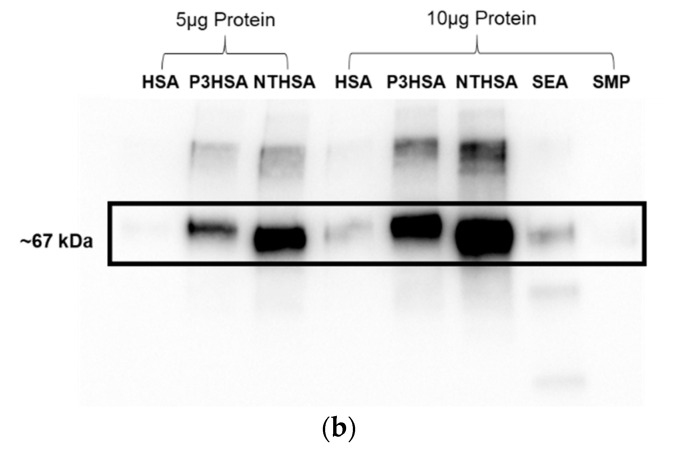
Binding of mAb F1P2H4D8D5 to sugar structures in ELISA and Western bolts. (**a**) mAb F1P2H4D8D5 was screened against sugar conjugates (P3DEX/P3HSA, NTDEX/NTHSA), carriers (DEX/HSA) and positive controls (SEA/SMP) via ELISA. F1P2H4D8D5 recognized P3DEX, P3HSA, and NTDEX via ELISA. (**b**) RAW 264.7 lysates treated with sugar conjugates (P3HSA/NTHSA) or carrier (HSA) were ran via SDS-PAGE, transferred to PVDF membranes, and probed with F1P2H4D8D5 via Western blot. F1P2H4D8D5 recognized both P3HSA and NTHSA via Western blot.

Next, we performed another screen of the purified mAb via ELISA against each of the sugar conjugates, the carrier structures (DEX and HSA), as well as against schisosome egg antigen (SEA) and schistosomula membrane protein (SMP). In this ELISA, our positive control mAb E.5 recognized P3DEX, P3HSA, SEA, and SMP (as expected) and did not bind to NTDEX or the carriers. The sole difference between LNFPIII and LNnT is the presence/absence of an α1,3-linked fucose residue, as depicted in the chemical structures shown in Figure 5a. The structure of the conjugates is given in Figure 5b, where 10–12 molecules of LNFPIII or LNnT are conjugated to DEX or HSA via the APD linker. Unexpectedly, purified mAb F1P2H4D8D5 recognized P3DEX and NTDEX, as well as P3HSA via ELISA (Figure 6a). Therefore, we initially believed that mAb F1P2H4D8D5 bound to a smaller structure within LNFPIII and LNnT. mAb F1P2H4D8D5 did not bind to DEX or HSA carriers, supporting this initial hypothesis. To further characterize mAb F1P2H4D8D5, RAW 264.7 cells were treated with HSA, P3HSA, or NTHSA. Lysates of the treated RAW 264.7 cells were prepared, run on an SDS-PAGE gel, and then transferred to a PVDF membrane, where they were probed with the mAb.

In Figure 6b, we found that, unlike proteins, the dextran conjugates did not run properly on an SDS-PAGE, nor could they be visualized. mAb F1P2H4D8D5 recognized both P3HSA and NTHSA on the Western blot, while it did not recognize NTHSA via ELISA. This led us to believe that the denaturing and reducing conditions generated via the use of β-mercaptoethanol and SDS-PAGE exposed specific epitopes on NTHSA, which allowed it to be recognized by mAb F1P2H4D8D5. Nonetheless, mAb F1P2H4D8D5 recognized both sugars in both conjugate forms. At this point, we altered our hypothesis, suggesting that mAb F1P2H4D8D5 potentially binds the APD linker of the conjugate.

**Figure 7:**
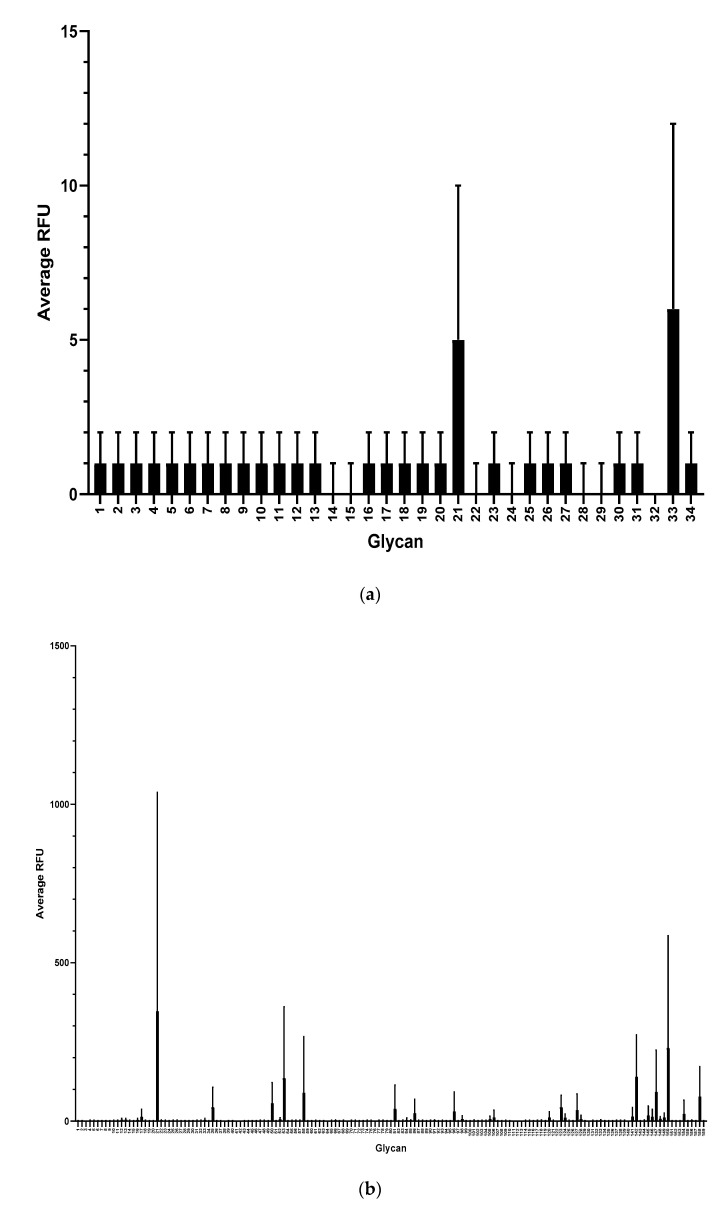
Screening of mAb F1P2H4D8D5 against glycan microarrays. (**a**) Human Milk Glycan Array and (**b**) NCFG Defined Glycan Array. Binding is depicted as average relative fluorescence units (RFU). Error bars represent standard deviation (SD).

To further characterize mAb F1P2H4D8D5 and determine if it recognized any common glycan structures, we sent aliquots of purified mAb to Harvard’s National Center for Functional Glycomics to be tested for binding on a Human Milk Glycan Microarray [31]. Surprisingly, mAb F1P2H4D8D5 did not bind to any of the glycans (1–34) present on the Human Milk Glycan Microarray (Figure 7a). mAb F1P2H4D8D5 was then tested for binding to the NCFG Defined Glycan Array (Figure 7b). This array contains various glycan structures, including the Lewis^x^ trisaccharide. Again, we were surprised to see that mAb F1P2H4D8D5 did not bind to any of the glycans (1–159) present on the array. This result supported our hypothesis that mAb F1P2H4D8D5 likely binds to the APD linker, since it did not recognize DEX (ELISA), HSA (ELISA/Western blot), or the sugar monomer/structures (LNnT, Lewis^x^).

## 4. Discussion

We attempted to produce mAbs binding to LNFPIII by immunizing mice with LNFPIII conjugates. We detected anti-LNFPIII conjugate antibodies in the sera of immunized mice after the prime. We boosted with the aim of generating IgG antibodies (Figure 1). Initial screens suggested that 3/75 hybridomas produced mAbs that bound to LNFPIII conjugates. It is important to note that the generation of antibodies to LNFPIII and other glycans is difficult. This is expected, as saccharide antigens are known to induce short-lived B cells that produce IgM antibodies independent of T cell help. This is in contrast to protein antigens, which stimulate long-lived B cells that produce IgG antibodies and lead to affinity maturation and memory B cells [18]. In this regard, we believe that the immunization of mice with LNFPIII conjugates, which present multiple LNFPIIIs in a manner that can cross-link cellular receptors, may be responsible for the observed anti-LNFPIII conjugate antibody responses, as B cells mature and develop an increased affinity for highly immunogenic antigens with multivalent displays. Unfortunately, despite a strong antibody binding to LNFPIII conjugates from the sera of immunized mice, mAb F1P2H4D8D5 did not bind to LNFPIII, but rather seemed to be specific for the APD linker spacer that was used to produce conjugates. We initially chose to clone F1P2H4, as it produced the highest IgG and IgM mAb responses. We have yet to screen in detail the other two hybridomas that were positive for binding to LNFPIII: F1P2C8 and F1P2D8 (Figure 2). However, we hypothesize that, as the majority of anti-Lewis^x^ antibodies are IgM and hybridoma F1P2D8 contained IgM mAbs, this hybridoma is more likely to be specific for LNFPIII (Figure 3). This could also be the case for clone F1P2H4D8A7 (Figure 3). Future studies will further investigate these hybridomas and clones.

The lack of available tools in glycan biology presents some important limitations. Based on our findings, we deduce that F1P2H4D8D5 binds to the linker spacer of the conjugate because it does not recognize the 40 kDa dextran or HSA carrier (Figure 6a,b) or any related glycan structure present on both the Human Milk Glycan Array (Figure 7a) or the NCFG Defined Glycan Array (Figure 7b) available via Harvard’s National Center for Functional Glycomics. While the arrays did not specifically contain LNFPIII, we were able to rule out that F1P2H4D8D5 did not bind to LNnT or LNFPI on the Human Milk Glycan Array. F1P2H4D8D5 also did not bind to the structures present on the NCFG Defined Glycan Array, including Lewis^x^. Taken together, these results further led to the conclusion that F1P2H4D8D5 binds to the linker spacer of the glycan conjugates.

Overall, this is important because mAb F1P2H4D8D5 recognized LNFPIII and LNnT conjugates internalized by RAW 264.7 cells. This mAb can be used as an immunological probe for ELISA, Western blot, immunoprecipitation, etc. to recognize conjugates that use this linker-spacer technology. Furthermore, it is important because the in vivo generation of antibodies to drug conjugates can result in drug clearance prior to therapeutic effects. While it is known that mAbs can be generated to the Lewis^x^ trisaccharide, there are few reports of antibodies specific to the HMOs, LNFPIII, or LNnT.

## 5. Conclusions

It is difficult to generate mAbs to carbohydrate moieties. We sought to produce mAbs to the HMO LNFPIII, but appear to have generated mAbs to the APD linker of LNFPIII and LNnT conjugates. We immunized with these conjugates, given that LNFPIII and LNnT do not appear to be antigenic in their free form. The conjugation to this APD linker allows the glycoconjugate to cross-link multiple receptors and bind multiple cell types [24,25,32,33,34,35,36]. LNFPIII conjugates have been shown to be therapeutic in murine models of inflammation-based conditions, such as psoriasis, multiple sclerosis, cardiac allograft survival, and nonalcoholic hepatosteatosis [26,37,38,39]. In this regard, it is important to investigate these conjugates further via the development of a mAb that can be used as a probe for mechanistic studies. This mAb would not only be useful for our studies, but also useful for determining the mechanism of other conjugates that utilize the APD linker.

## Figures and Tables

**Table 1:** mAb Purification Table. Protein concentrations and 260/280 ratios following each purification step.

Purification Step	Protein [mg/mL]	260/280 Ratio
Pre-Ammonium Sulfate	4.93	0.83
Post-25% Ammonium Sulfate	3.76	0.83
Post-50% Ammonium Sulfate	3.19	0.96
Post-Dialysis	2.64	0.85
Post-G Column Purification	1.79	0.57

## References

[1] Gao C., Wei M., McKitrick T.R., McQuillan A.M., Heimburg-Molinaro J., Cummings R.D. (2019). Glycan microarrays as chemical tools for identifying glycan recognition by immune proteins. Front. Chem..

[2] Varki A. (2016). Biological roles of glycans. Glycobiology.

[3] Copoiu L., Malhotra S. (2020). The current structural glycome landscape and emerging technologies. Curr. Opin. Struct. Biol..

[4] Haab B.B., Klamer Z. (2020). Advances in tools to determine the glycan-binding specificities of lectins and antibodies. Mol. Cell. Proteom..

[5] Nakamura-Tsuruta S., Kominami J., Kuno A., Hirabayashi J. (2006). Evidence that Agaricus bisporus agglutinin (ABA) has dual sugar-binding specificity. Biochem. Biophys. Res. Commun..

[6] Matsumura K., Higashida K., Hata Y., Kominami J., Nakamura-Tsuruta S., Hirabayashi J. (2009). Comparative analysis of oligosaccharide specificities of fucose-specific lectins from Aspergillus oryzae and Aleuria aurantia using frontal affinity chromatography. Anal. Biochem..

[7] Liu X., Lei Z., Liu D., Wang Z. (2016). Development of a sandwiched microarray platform for studying the interactions of antibiotics with Staphylococcus aureus. Anal. Chim. Acta.

[8] Hsu K.-L., Pilobello K.T., Mahal L.K. (2006). Analyzing the dynamic bacterial glycome with a lectin microarray approach. Nat. Chem. Biol..

[9] Kilcoyne M., Twomey M.E., Gerlach J.Q., Kane M., Moran A.P., Joshi L. (2014). Campylobacter jejuni strain discrimination and temperature-dependent glycome expression profiling by lectin microarray. Carbohydr. Res..

[10] Yasuda E., Tateno H., Hirabarashi J., Iino T., Sako T. (2011). Lectin microarray reveals binding profiles of lactobacillus casei strains in a comprehensive analysis of bacterial cell wall polysaccharides. Appl. Environ. Microbiol..

[11] Dang K., Zhang W., Jiang S., Lin X., Qian A. (2020). Application of lectin microarrays for biomarker discovery. ChemistryOpen.

[12] Cummings R.D., Darvill A.G., Etzler M.E., Hahn M.G., Varki A., Cummings R.D., Esko J.D., Stanley P., Hart G.W., Aebi M., Darvill A.G., Kinoshita T., Packer N.H., Prestegard J.H. (2015). Glycan-recognizing probes as tools. Essentials of Glycobiology.

[13] Mandalasi M., Dorabawila N., Smith D.F., Heimburg-Molinaro J., Cummings R.D., Nyame A.K. (2013). Development and characterization of a specific IgG monoclonal antibody toward the Lewis x antigen using splenocytes of Schistosoma mansoni-infected mice. Glycobiology.

[14] Harn D.A., Mitsuyama M., David J.R. (1984). Schistosoma mansoni. Anti-egg monoclonal antibodies protect against cercarial challenge in vivo. J. Exp. Med..

[15] Ko A.I., Harn D.A. (1987). Characterization of protective and non-protective surface membrane carbohydrate epitopes of Schistosoma mansoni. Mem. Inst. Oswaldo Cruz.

[16] Harn D.A., Quinn J.J., Cianci C.M., Ko A.I. (1987). Evidence that a protective membrane epitope is involved in early but not late phase immunity in Schistosoma mansoni. J. Immunol..

[17] Ko A.I., Drager U.C., Harn D.A. (1990). A Schistosoma mansoni epitope recognized by a protective monoclonal antibody is identical to the stage-specific embryonic antigen 1. Proc. Natl. Acad. Sci. USA.

[18] Heimburg-Molinaro J., Rittenhouse-Olson K., Packer N.H., Karlsson N.G. (2009). Development and characterization of antibodies to carbohydrate antigens. Glycomics: Methods and Protocols.

[19] Solter D., Knowles B.B. (1978). Monoclonal antibody defining a stage-specific mouse embryonic antigen (SSEA-1). Proc. Natl. Acad. Sci. USA.

[20] Hokke C.H., Deelder A.M. (2001). Schistosome glycoconjugates in host-parasite interplay. Glycoconj. J..

[21] Smilowitz J.T., Lebrilla C.B., Mills D.A., German J.B., Freeman S.L. (2014). Breast milk oligosaccharides: Structure-function relationships in the neonate. Annu. Rev. Nutr..

[22] Palanivel V., Posey C., Horauf A.M., Solbach W., Piessens W.F., Harn D.A. (1996). B-cell outgrowth and ligand-specific production of IL-10 correlate with Th2 dominance in certain parasitic diseases. Exp. Parasitol..

[23] Atochina O., Daly-Engel T., Piskorska D., McGuire E., Harn D.A. (2001). A schistosome-expressed immunomodulatory glycoconjugate expands peritoneal Gr1(+) macrophages that suppress naive CD4(+) T cell proliferation via an IFN-gamma and nitric oxide-dependent mechanism. J. Immunol..

[24] Thomas P.G., Carter M.R., Atochina O., Da’Dara A.A., Piskorska D., McGuire E., Harn D.A. (2003). Maturation of dendritic cell 2 phenotype by a helminth glycan uses a Toll-like receptor 4-dependent mechanism. J. Immunol..

[25] Thomas P.G., Carter M.R., Da’dara A.A., DeSimone T.M., Harn D.A. (2005). A helminth glycan induces APC maturation via alternative NF-kappa B activation independent of I kappa B alpha degradation. J. Immunol..

[26] Bhargava P., Li C., Stanya K.J., Jacobi D., Dai L., Liu S., Gangl M.R., Harn D.A., Lee C.H. (2012). Immunomodulatory glycan LNFPIII alleviates hepatosteatosis and insulin resistance through direct and indirect control of metabolic pathways. Nat. Med..

[27] Tivadar S.T., McIntosh R.S., Chua J.X., Moss R., Parsons T., Zaitoun A.M., Madhusudan S., Durrant L.G., Vankemmelbeke M. (2020). Monoclonal antibody targeting sialyl-di-Lewisa–containing internalizing and noninternalizing glycoproteins with cancer immunotherapy development potential. Mol. Cancer Ther..

[28] De Rougemont A., Ruvoen-Clouet N., Simon B., Estienney M., Elie-Caille C., Aho S., Pothier P., Le Pendu J., Boireau W., Belliot G. (2011). Qualitative and quantitative analysis of the binding of GII.4 norovirus variants onto human blood group antigens. J. Virol..

[29] Grenfell R.F., Shollenberger L.M., Samli E.F., Harn D.A. (2015). Vaccine self-assembling immune matrix is a new delivery platform that enhances immune responses to recombinant HBsAg in mice. Clin. Vaccine Immunol..

[30] Fishman J.B., Berg E.A. (2018). Ammonium sulfate fractionation of antibodies. Cold Spring Harb. Protoc..

[31] Yu Y., Mishra S., Song X., Lasanajak Y., Bradley K.C., Tappert M.M., Air G.M., Steinhauer D.A., Halder S., Cotmore S. (2012). Functional glycomic analysis of human milk glycans reveals the presence of virus receptors and embryonic stem cell biomarkers. J. Biol. Chem..

[32] Velupillai P., Harn D.A. (1994). Oligosaccharide-specific induction of interleukin 10 production by B220+ cells from schistosome-infected mice: A mechanism for regulation of CD4+ T-cell subsets. Proc. Natl. Acad. Sci. USA.

[33] Atochina O., Harn D. (2005). LNFPIII/LeX-stimulated macrophages activate natural killer cells via CD40-CD40L interaction. Clin. Diagn. Lab. Immunol..

[34] Atochina O., Da’dara A.A., Walker M., Harn D.A. (2008). The immunomodulatory glycan LNFPIII initiates alternative activation of murine macrophages in vivo. Immunology.

[35] Srivastava L., Tundup S., Choi B.S., Norberg T., Harn D. (2014). Immunomodulatory glycan lacto-N-fucopentaose III requires clathrin-mediated endocytosis to induce alternative activation of antigen-presenting cells. Infect. Immun..

[36] Tundup S., Srivastava L., Nagy T., Harn D. (2014). CD14 influences host immune responses and alternative activation of macrophages during Schistosoma mansoni infection. Infect. Immun..

[37] Atochina O., Harn D. (2006). Prevention of psoriasis-like lesions development in fsn/fsn mice by helminth glycans. Exp. Dermatol..

[38] Zhu B., Trikudanathan S., Zozulya A.L., Sandoval-Garcia C., Kennedy J.K., Atochina O., Norberg T., Castagner B., Seeberger P., Fabry Z. (2012). Immune modulation by Lacto-N-fucopentaose III in experimental autoimmune encephalomyelitis. Clin. Immunol..

[39] Dutta P., Hullett D.A., Roenneburg D.A., Torrealba J.R., Sollinger H.W., Harn D.A., Burlingham W.J. (2010). Lacto-N-fucopentaose III, a pentasaccharide, prolongs heart transplant survival. Transplantation.

